# Fabrication and In Vitro Evaluation of LL37-Loaded Electrospun PHB/Collagen Nanofibers for Wound Healing

**DOI:** 10.3390/polym17182486

**Published:** 2025-09-15

**Authors:** Beyza Nur Sayaner Taşçı, Sümeyye Kozan, Meltem Demirel Kars, Kemal Çetin, Sema Karslıoğlu, Gökhan Kars

**Affiliations:** 1 Department of Molecular Biology and Genetics, Faculty of Science, Necmettin Erbakan University, Konya 42140, Türkiye; 2 Department of Biomedical Engineering, Faculty of Engineering, Necmettin Erbakan University, Konya 42140, Türkiye; sumeyyekozan@ogr.erbakan.edu.tr (S.K.); mdkars@erbakan.edu.tr (M.D.K.); 3 Department of Biotechnology, Faculty of Science, Necmettin Erbakan University, Konya 42140, Türkiye; kcetin@erbakan.edu.tr; 4 Department of Basic Sciences, Faculty of Engineering, Necmettin Erbakan University, Konya 42140, Türkiye; svural@erbakan.edu.tr

**Keywords:** biofiber, collagen, LL37, PHB, wound healing

## Abstract

Skin repair is essential in the treatment of burns and wounds. After an injury, the concept of tissue engineering emerges to restore skin function and facilitate wound healing. This field often involves the use of biodegradable and biocompatible materials as a primary scaffold for tissue regeneration. In this study, a PHB/Collagen wound dressing mat loaded with the antimicrobial peptide LL37 was developed via electrospinning. The polymer solutions were prepared by dissolving polyhydroxybutyrate (PHB) biopolymer extracted from *Cereibacter sphaeroides*, commercial PHB, and marine collagen in hexafluoroisopropanol (HFIP). The resulting nanofibers were characterized using Field-Emission Scanning Electron Microscopy (FE-SEM), Thermogravimetric Analysis (TGA), X-Ray Diffractometry (XRD), and an Optical Tensiometer. Antibacterial activity assessments were conducted against *Staphylococcus aureus* (ATCC 29213) and *Escherichia coli* (ATCC 25922). Degradability studies were carried out in DMEM medium, cytotoxicity tests were performed on the L929 fibroblast cell line, and the wound healing effect was investigated on the HS2 keratinocyte cell line. To evaluate the properties of the designed material under in vitro conditions, the morphology of cells on the nanofiber was examined using an inverted light microscope. The findings demonstrated that the nanofibers were biocompatible in vitro and exhibited no toxic effects. And, compared to the control groups, the 5.56 nmol LL37-loaded PHB/Collagen nanofibers significantly enhanced wound closure by 15–30% and effectively reduced the viability of *S. aureus* and *E. coli* by 20–25% and approximately 80–85%, respectively. These results highlight the therapeutic potential of LL37-loaded PHB/Collagen nanofibers for use in wound healing applications.

## 1. Introduction

Polymers are high-molecular-weight macromolecules formed by the association of repeating monomer units with covalent bonds. Based on their sources and biological properties, they are classified as natural polymers (e.g., polysaccharides and proteins), biodegradable synthetic polymers (esters, amides, ethers, urethanes), and hybrid systems [1]. Biopolymers, among these groups, are noteworthy materials in biomedical applications due to their natural origin, biodegradable structure, and biocompatible properties. Biopolymers are generally divided into three main categories: (i) biopolymers obtained from agricultural sources such as starch, (ii) polymers such as polyhydroxyalkanoates (PHA) obtained through microbial activities, and (iii) polymers such as polylactic acid (PLA) synthesized through biotechnological means [2].

Wound healing is a clinically important process, particularly in the treatment of chronic wounds and burns. Such wounds increase the risk of infection, prolong the healing process, and pose a significant economic burden on healthcare systems [3]. Therefore, the need for biomaterial systems that accelerate wound healing, prevent infections, and support tissue repair is increasing. Tissue engineering is gaining prominence in this context, and structures that support cell growth are being developed using biodegradable and biocompatible scaffolding materials [4].

Collagen, one of the natural polymers, is the most abundant structural protein in the extracellular matrix. Its advantages, such as low antigenicity, biocompatibility, and biodegradability, make it widely used in wound dressings. Collagen supports fibroblast proliferation, promotes endothelial cell migration, stimulates coagulation, and contributes to scar formation [3]. These properties make collagen a frequently preferred natural biopolymer in wound healing. Furthermore, polyhydroxybutyrate (PHB), a microbially derived biopolymer, is a member of the PHA family and has also biodegradable and biocompatible structures. Its potential for natural interaction with human blood and tissues makes it an attractive material for biomedical applications [5]. PHB synthesis generally occurs through the metabolism of carbon sources such as glucose by microorganisms. Bacteria such as *Bacillus*, *Pseudomonas*, *Klebsiella*, *Streptomyces*, *Mycobacterium*, *Rhodococcus*, and *Escherichia* play a significant role in PHB production [6].

Today, the development of antimicrobial biomaterials to prevent wound infections is attracting considerable interest. Antimicrobial peptides (AMPs) exhibit broad-spectrum antimicrobial activity by strongly interacting with bacterial cell membranes due to their positively charged and hydrophobic structures [7]. These peptides, which can be physically or chemically immobilized on nanofiber surfaces, have the potential to replace traditional antibiotics [8]. One of these peptides, LL37, is a positively charged molecule with an α-helical structure and exhibits strong antibacterial activity under physiological conditions such as pH 6. LL37 provides antimicrobial activity by disrupting the integrity of the bacterial membrane. However, it also has limitations such as low biological stability, sensitivity to proteolytic enzymes, and potential cytotoxicity [7].

In the literature, most studies on PHB have primarily focused on comparing the yield and purity obtained after fermentation using different bacterial strains. However, beyond yield, it is also well established that the chemical, mechanical, thermal, and crystalline characteristics of PHB vary significantly depending on the producing strain, fermentation conditions, and extraction procedure [9]. For instance, bacterial PHB exhibited a lower molar mass compared to commercial PHB, which in turn influences crystallinity, thermal stability, and processability. This indicates that the properties of PHB are not universal but rather context-dependent. Mixing PHB from different origins, such as commercially available PHB and PHB extracted from *Cereibacter sphaeroides*, allows tailoring of material properties that cannot be achieved with a single source. Commercial PHB, with a higher molecular weight (~500 kDa), provides consistency in purity and thermal behavior, while PHB from *C. sphaeroides* (~102 kDa) contributes unique strain-specific characteristics, including lower crystallinity, altered mechanical behavior, and increased hydrophilicity. By blending these polymers, it is possible to produce fibers with finer morphology, enhanced hydrophilic properties, controlled biodegradability, and tunable thermal and structural characteristics. This approach offers a flexible strategy to optimize the performance of PHB-based materials for biomedical and other applications.

In our study, we utilized PHB produced under specific fermentation and extraction conditions, resulting in a material with unique structural and functional characteristics [10]. By integrating this distinct PHB source with marine collagen and functionalizing the electrospun scaffold with LL37, we addressed a gap in the literature, since such a combination has not been systematically explored before. This unique integration highlights the novelty of our work and differentiates it from previous PHB/Collagen/AMP systems reported in the literature. In the current study, multifunctional biocompatible and antibacterial wound dressings were designed by integrating the LL37 peptide into an electrospun biofiber mat containing commercial and bacterial PHB and collagen The structural characterization, antibacterial activity, and cellular biocompatibility of the developed system were thoroughly evaluated in vitro. The findings demonstrate the potential of this next-generation biomaterial platform for wound healing.

## 2. Materials and Methods

### 2.1. Chemicals and Materials

PHB extracted from *Cereibacter sphaeroides* O.U.001, commercially available PHB (500 kDa, Merck, Darmstadt, Germany), marine-derived collagen, LL37 antimicrobial peptide (QYABIO, Sigma-Aldrich, St. Louis, MO, USA), 1,1,1,3,3,3-Hexafluoro-2-propanol (HFIP) (Abcr, Karlsruhe, Germany), Dulbecco’s Modified Eagle Medium (DMEM) (Gibco, Grand Island, NY, USA), fetal bovine serum (FBS), gentamicin (Sigma-Aldrich, St. Louis, MO, USA), and XTT (3-(4,5-dimethylthiazol-2-yl)-2,5-diphenyltetrazolium bromide assay, Sartorius, Göttingen, Germany) were purchased.

### 2.2. Method

#### 2.2.1. Development of LL37-Loaded PHB/Col Nanofiber Mats

**Figure 1:**
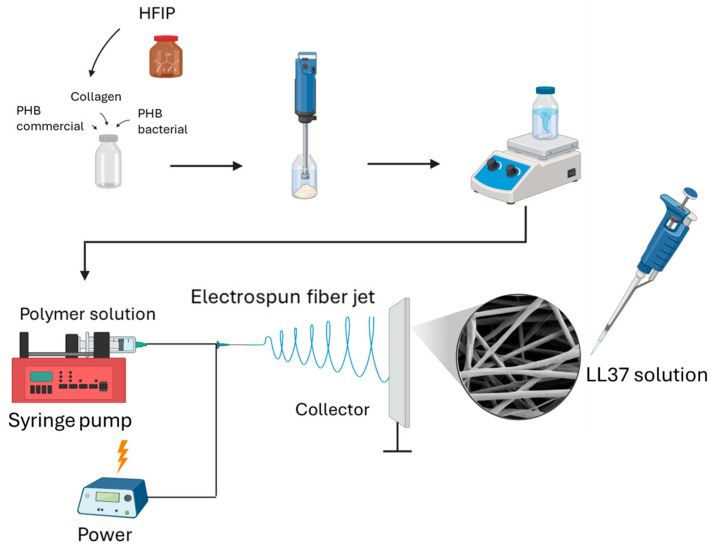
Schematic representation of PHB/Col/LL37 nanofibers.

Bacterial PHB, commercial PHB, and marine collagen were utilized as polymeric components. Bacterial PHB was incorporated at varying percentages of the total PHB content, i.e., 0%, 25%, 50%, 75%, and 100% (*w*/*w*). Nanofibers were prepared according to a previously described method with some modifications [11]. For each formulation, 20 mL of polymer solutions was prepared, maintaining a total polymer concentration of 2% (*w*/*v*) with a PHB/Collagen ratio of 1:1 (*w*/*w*) (Table 1). These solutions were sonicated using a Bandelin Sonopuls HD 2200 MS 72 (Bandelin Electronic, Berlin, Germany) in an icebox for 10 min and homogenized by overnight stirring using a magnetic stirrer to ensure complete dissolution and uniform dispersion. The polymer solutions were transferred into 10 mL syringes and electrospun onto aluminum foil collectors using a syringe pump. Optimized electrospinning parameters (a flow rate of 1.8 mL/h, a working distance of 18 cm, and an applied voltage of 27 kV) were employed to produce nanofiber wound dressing prototypes. The PHB/Collagen nanofibers were loaded with the antimicrobial peptide LL37 via a physical adsorption method because covalent binding carries the risk of permanently altering the peptide’s delicate three-dimensional structure and compromising its biological activity. The strong covalent bonding could also negatively impact the rate of therapeutic release [11]. Besides these, the noncovalent approach also offers a simple drug-loading process [12]. Specifically, 25 µL of LL37 solution (1 mg/mL) was precisely dripped onto circular nanofiber samples (0.6 cm in diameter) and allowed to air dry (Figure 1).

#### 2.2.2. Physicochemical Characterization of LL37/PHB/Col Nanofiber Mats

The morphological characteristics of PHB/Collagen nanofiber mats were analyzed using a field-emission scanning electron microscope (FE-SEM) (Gemini SEM 500, Zeiss, Oberkochen, Germany). The samples were examined under an accelerating voltage of 2 kV to ensure high-resolution imaging. The fiber diameters were measured and analyzed by the ImageJ 1.53t program and the fiber diameters were presented on histogram graphs. The wettabilities of the nanofiber mats were tested by digital video-based optical contact angle measurement using a Biolin Scientific Attension Theta Lite instrument (Biolin Scientific, Göteborg, Sweden).

The chemical characterization was performed by common methods: FT-IR, XRD, and thermogravimetric analyses. FT-IR analysis was used to identify specific functional groups present in the chemical bonds of fiber materials; X-ray diffraction (XRD) analysis was conducted to reveal the distribution and crystalline structure of the nanofibers; and thermogravimetric analysis (TGA, Setaram, Labsys Evo, Caluire, France) was performed to determine the mass loss of the nanofibers in response to temperature changes.

#### 2.2.3. Degradation of Nanofibers

In this section, the degradation of the biomaterial in biological fluid over a 14-day period is evaluated in terms of weight loss. The experiments were conducted in a cell culture medium supplemented with serum at a neutral pH (DMEM). No enzymes or bacteria were introduced into the degradation medium. In vitro degradability testing of PHB/Collagen wound dressing functionalized with LL37 antimicrobial peptide was carried out to obtain information about the durability, efficacy, and safety of the nanofiber material under biologically relevant conditions. In this context, nanofiber mat samples were cut into 0.6 cm diameter discs with the help of a punch. The discs were placed in 96-well microplates in triplicate and 150 µL DMEM medium was added to each well. Nanofiber discs were removed from the medium on days 1, 7, and 14, and were dried and weighed. Based on the data, the weight loss percentage of the samples was calculated, and the physical degradability profile of the nanofibers was evaluated in terms of weight loss in biological fluid.

#### 2.2.4. In Vitro Biocompatibility and Cell Attachment on Nanofibers

The in vitro biocompatibility of PHB/Collagen NF wound dressing samples was assessed by evaluating their effects on the proliferation of mouse fibroblast L929 cells. NFs were incubated in a blank medium (L929, DMEM) for 24, 48, and 72 h. At the end of these periods, nanofibers were removed from the medium, which were tested for biocompatibility. Then, 5 × 10^3^ L929 cells per well were added to each well of the 96-well microplate, except for the negative control and medium control, and the cells were incubated for 24 h for attachment. At the end of the incubation period, the medium was removed, and the nanofiber extracts were transferred to the wells. The extract-treated cells were visualized with an inverted light microscope (10× magnification), then 50 μL of XTT solution was added to each well (500 μL of XTT prepared with 50 μL of activator), and the plates were incubated for 4 h. Cell proliferation was assessed by optical density (OD) measurements at 450, 500, and 630 nm wavelengths using a microplate spectrophotometer. The data were used to evaluate the effect of nanofibers on cell viability. The cell viability percentage of L929 cells was calculated using Equation (1).

(1) $$Cell\;viability\;\left(\%\right)=\frac{The\;optical\;density\;\left(OD\right)\;450\;nm\;of\;the\;sample}{The\;OD\;450\;nm\;of\;the\;positive\;control-The\;OD\;450\;nm\;of\;the\;negative\;control}\times 100$$

The biocompatibility of the LL37 AMP-loaded PHB/Collagen nanofiber wound dressing mats and cell attachment ability on NF/LL37 mats were analyzed by a confocal laser scanning microscope (CLSM) (Zeiss, Oberkochen, Germany) and a scanning electron microscope (SEM, Hitachi High-Tech Corp., Tokyo, Japan) to evaluate cell adhesion.

The nanofibers were punched into 0.6 cm diameter discs and sterilized on both sides under UV light for 30 min. They were then placed into 96-well cell culture plates and rinsed twice with PBS. Keratinocytes cells were seeded at 1.5 × 10^5^ cell density in DMEM medium (supplemented with 70% FBS and gentamicin) onto each nanofiber scaffold. After allowing the cells to adhere to the nanofiber surface, the samples were rinsed with 0.1 M sodium cacodylate buffer (pH 7.4) and fixed by immersion in 3% glutaraldehyde. For confocal microscopy after fixation, fibers were rinsed with 300 µL of Tween-80 solution, and the fibers were treated with Triton-X solution for 5 min. The staining solution (rhodamine–phalloidin and DAPI) was applied to the fibers, followed by incubation for 20 min. Subsequently, the samples were washed with Tween-80 and then prepared for confocal microscopy by adding 1 mL of PBS. For SEM analysis, following fixation, the samples were washed with distilled water and dehydrated in ethanol solutions (50%, 75%, 90%, and 100% *v*/*v*). The dehydrated samples were then treated with HMDS and dried overnight in a fume hood. Finally, the samples were coated with iridium and analyzed under SEM.

#### 2.2.5. Measurement of 2D Wound Healing Activity by Scratch Assay

The in vitro wound healing effects of the NF/LL37 samples were evaluated using the scratch assay technique. In this study, 0.4 × 10^5^ human keratinocyte cells were seeded into each well of a 96-well microplate and incubated to allow the cells to cover the surface. A scratch was made in each well using a sterile 100 µL pipette tip. The microplate was divided into two groups with three replicates each, NF/LL37 samples and blank NF samples, and the corresponding nanofiber samples were placed over the scratches. On days 1, 3, and 7, the nanofibers were removed, and the scratch closure was observed under an inverted light microscope (4× magnification). The images were analyzed using ImageJ software 1.53t to measure the scratch closure rates and evaluate the wound healing potential. The scratch closure percentage was calculated according to Equation (2), where A_0_ is the initial scratch width and A_t_ is the scratch width at a given time.

(2) $$Scratch\;closure\;\left(\%\right)=\frac{A_{0}-A_{t}}{A_{0}}\times 100$$

#### 2.2.6. Evaluation of Antibacterial Activity of Nanofiber Mats

The in vitro antibacterial activity of NF mats was evaluated against *Escherichia coli* (ATCC 25922) and *Staphylococcus aureus* (ATCC 29213). For this purpose, bacterial strains were cultured on Mueller Hinton Agar (MHA) at 37 °C for 16 h. Nanofiber discs with a diameter of 0.6 cm were sterilized under UV light, after which 25 µL of LL37 solution (1 mg/mL) was dropped onto each disc and left to dry under sterile conditions. Bacterial suspensions were adjusted to the 0.5 McFarland standard. Each well received 90 µL of Mueller Hinton Broth (MHB), and 10 µL of the prepared bacterial suspension was added to the wells, and NF/LL37 nanofiber discs were placed on bacteria suspension, in triplicate. For the negative control group, Gentamicin and Oxacillin antibiotic discs were placed into the wells. The non-treated bacteria suspension was served as positive control for cell viability. The plate was incubated at 37 °C for 16 h. After incubation, optical density (OD) measurements at 600 nm were performed using a 96-well-plate reader to assess antibacterial activity. These measurements were used to determine the inhibitory effects of the nanofibers on bacterial growth.

## 3. Results and Discussion

### 3.1. Physicochemical Characteristics of PHB/Col/LL37 Nanofiber Mats

**Figure 2:**
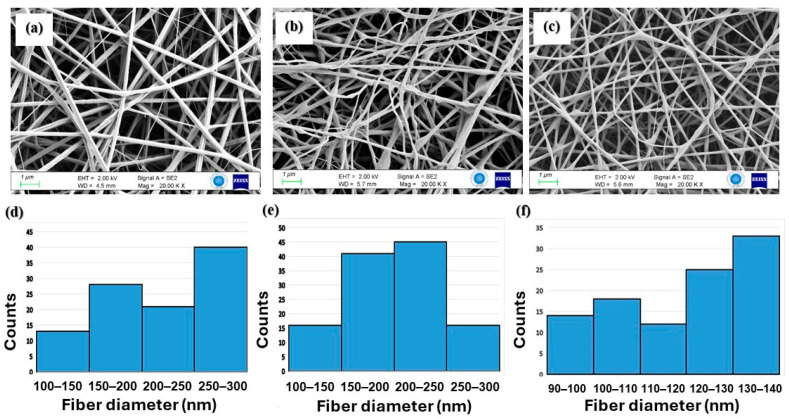
FE-SEM images of PHB/Col nanofibers and distribution of fiber diameters of NF-1 (**a**,**d**), NF-2 (**b**,**e**), and NF-3 (**c**,**f**).

The electrospun nanofibers manufactured with different PHB/Collagen ratios were tested by Salvatore et al., and it was found that increasing the collagen content (1:1 ratio) affected both viscosity and fiber diameter due to increased solution viscosity [13]. Similarly, Prabhakaran et al. reported an inverse effect of collagen content on fiber size in PHBV/Collagen blends. Accordingly, a PHB/Collagen ratio of 1:1 was adopted in this study [14]. Among the six different PHB_b_/PHB_c_-containing polymer slurries, the ones containing 0%, 25%, and 50% bacterial PHB resulted in successfully spun nanofibers (Figure 2). Nanofiber formation was not observed with the 75% and 100% PHB_b_/Collagen solutions (NF-4 and NF-5). This may have been because of the very low dissolution of bacterial PHB at higher ratios, which may have stemmed from low-molar-mass PHB_b_, resulting in a turbid, low-viscosity solution.

A detailed FE-SEM analysis at 20.00 KX magnification revealed a uniform, bead-free network structure of the nanofibers. Further analysis using ImageJ 1.53t enabled estimation of average fiber diameter and distribution for each membrane type. It is well known that viscosity significantly affects jet formation and fiber thinning during electrospinning. A lower viscosity typically yields finer fibers. In this study, increasing the bacterial PHB content reduced solution viscosity, resulting in more uniform and finer fibers (Figure 2a–c). The mean fiber diameters of NF-1, NF-2, and NF-3 were found to be 232.80 ± 62.04 nm, 212.64 ± 53.73 nm, and 119.82 ± 18.25 nm, respectively (Figure 2d–f). The morphology of LL37-loaded nanofibers were presented by FE-SEM analysis as a Supplementary Materials (Figure S1).

**Figure 3:**
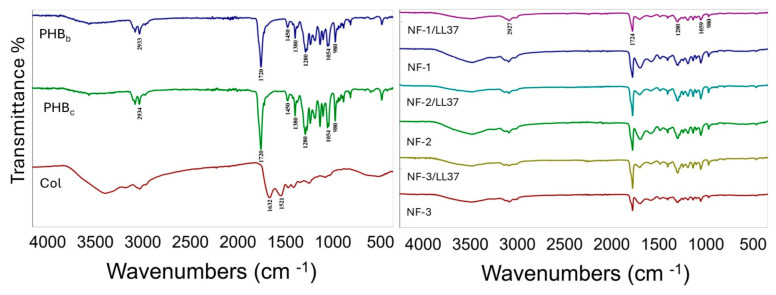
FT-IR spectra of raw materials of polymer slurry (PHB_c_, PHB_b_, Col) and that of NF mats.

FT-IR analysis was performed to identify the functional groups of PHB extracted from *Cereibacter sphaeroides*, as well as commercial PHB and collagen obtained from Sigma Aldrich (St. Louis, MO, USA). Functional groups of PHB were examined by comparing characteristic peaks at specific wavelengths. The FT-IR spectra of bacterial and commercial PHB, along with marine collagen, were recorded between 4000 and 500 cm^−1^ (Figure 3), and the corresponding functional groups are listed in Table 2.

The FT-IR spectrum of PHB showed characteristic peaks confirming the presence of polyhydroxybutyrate in the nanofibers. As expected, the polymer exhibited a strong C=O peak, verifying its identity as PHB [15]. As shown in Table 2 and Figure 3, absorption at 2976–2932 cm^−1^ indicated aliphatic CH stretching, while the peak at 1724–1719 cm^−1^ corresponded to the C=O stretching vibration of ester groups. Additional peaks at 1453 cm^−1^ (CH_2_ scissoring), 1380 cm^−1^ (CH_3_ bending), and 1275–1280 cm^−1^ (C–O stretching) further confirmed the structure. In line with Prabhakaran et al., collagen-specific amide I and II peaks were observed at 1632 cm^−1^ and 1521 cm^−1^, validating the presence of marine-derived collagen in the NF samples [14].

**Figure 4:**
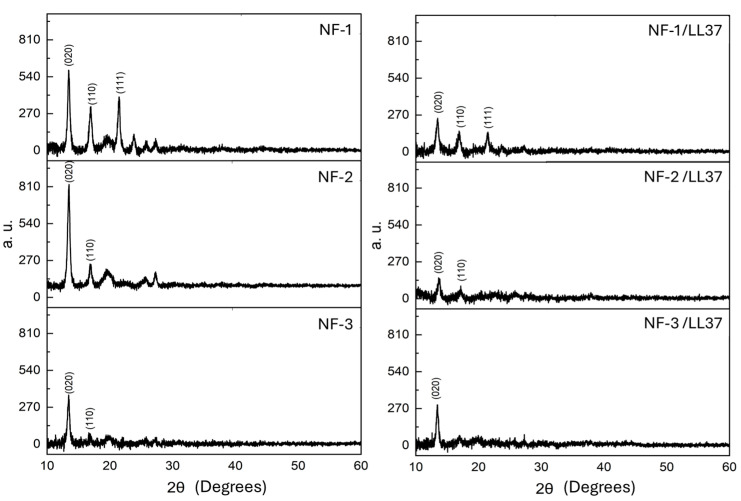
XRD spectra of NFs.

The decrease in crystallinity observed with increasing bacterial PHB content and the incorporation of LL37 aligns with previously reported trends in similar PHB-based systems. As shown in Figure 4, distinct diffraction peaks near 2θ ≈ 13°, 17°, and 22°, corresponding to the orthorhombic crystalline planes (020), (110), and (111) of PHB, are most prominent in the NF-1 sample, which contains only high-molecular-mass commercial PHB (~500 kDa). This indicates high crystallinity arising from regular chain packing. In contrast, the crystallinity decreases markedly in the NF-2 and NF-3 samples with the addition of bacterial PHB (PHB_b_, ~102 kDa). This trend is consistent with earlier studies demonstrating that lower molecular mass and structural irregularities in bacterial PHB disrupt ordered crystalline domains and hinder spherulite formation [16,17].

The incorporation of LL37 further disrupts crystallinity, as evidenced by the weakening and broadening of diffraction peaks, particularly in the NF-2/LL37 and NF-3/LL37 samples, where some reflections nearly disappear. This behavior resembles classical plasticization phenomena, wherein low-molecular-mass additives interfere with polymer chain packing, as reported for various plasticizers in PHB and PHBV systems [18,19]. The amphiphilic nature and low molecular mass of LL37 (~4.5 kDa) likely enable it to diffuse between PHB chains, weakening secondary interactions and reducing the continuity of crystalline domains. As a result, polymer chain mobility increases. This enhanced mobility facilitates fiber spinning and contributes to the formation of finer nanofibers, in agreement with previous approaches that improved PHB processability via internal or external modifiers [20].

According to the TGA results, all nanofiber composites exhibit a major weight loss event in the range of 280–310 °C, corresponding to the primary thermal decomposition of the PHB phase. The onset temperature and degradation rate vary with bacterial PHB (PHB_b_) content. NF-3 (50% PHB_b_) shows the earliest degradation, followed by NF-2 (25% PHB_b_) and NF-1 (0% PHB_b_). This behavior is attributed to the lower molar mass and shorter chain length of PHB_b_, which increase the number of chain ends and structural irregularities, making it more susceptible to thermal scission. In contrast, NF-1, containing only commercial PHB (PHB_c_), displays delayed weight loss and greater thermal stability due to its higher molar mass and crystallinity.

The degradation curves further reveal that higher PHB_b_ contents lead to steeper mass losses and lower char yields at 600 °C. Residue analysis confirms this trend: NF-1 retains the highest char fraction, while NF-2 and NF-3 show progressively lower residues. These results suggest that PHB_b_ disrupts the crystalline packing of PHB_c_, accelerates volatilization, and reduces carbonization due to the formation of low-molecular-mass, volatile products. Although collagen may contribute to the residual mass, the PHB_b_/PHB_c_ ratio is the dominant factor determining thermal stability.

Overall, incorporation of PHB_b_ not only shifts degradation onset to lower temperatures but also alters the decomposition mechanism by reducing crystallinity, lowering char formation, and promoting more volatile degradation products. This highlights the crucial role of molar mass distribution and chain regularity in governing the thermal behavior of PHB-based nanofibers. DSC data support these findings. All samples exhibit PHB-related melting peaks around 175–180 °C, but the peak intensity decreases with higher PHB_b_ content. NF-1 shows a sharp, high-enthalpy peak, indicating high crystallinity, while NF-2 and NF-3 samples show broader, weaker peaks, reflecting disrupted crystal formation. Broad exothermic transitions between 300 and 500 °C are linked to collagen degradation and internal oxidation, and these transitions diminish with increasing PHB_b_ content.

**Figure 5:**
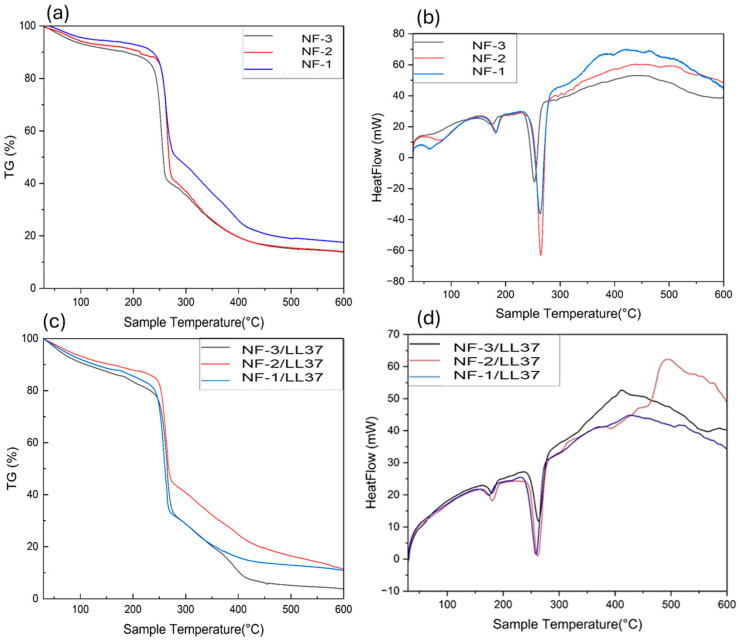
Thermal properties of PHB/Collagen samples containing LL37 and varying ratios of bacterial PHB (PHB_b_). (**a**,**c**) Thermogravimetric analysis (TGA) curves; (**b**,**d**) differential scanning calorimetry (DSC) curves.

Overall, higher bacterial PHB content reduces crystallinity and thermal stability, shifting the material toward a more amorphous structure. This is consistent with an earlier onset of degradation in TGA and weaker melting peaks in DSC. While PHB_b_ enhances processability, it compromises crystallinity and thermal resistance. Figure 5 illustrates how LL37 and varying PHB_b_ ratios affect thermal behavior in PHB/Collagen samples. TGA confirms degradation onset shifts earlier with increasing PHB_b_, especially at 50%. Residual mass decreases significantly at high PHB_b_ content. DSC results show diminishing and broadening melting peaks with increased PHB_b_, reflecting greater amorphous character. Broad exothermic transitions between 300 and 500 °C are primarily due to collagen pyrolysis, with the 25% PHB_b_ sample showing the most pronounced response, possibly due to synergistic degradation of collagen and peptide interactions. In conclusion, increasing PHB_b_ reduces crystallinity and thermal stability, and LL37 acts as a secondary plasticizing agent, disrupting chain packing and enhancing matrix degradation.

**Figure 6:**
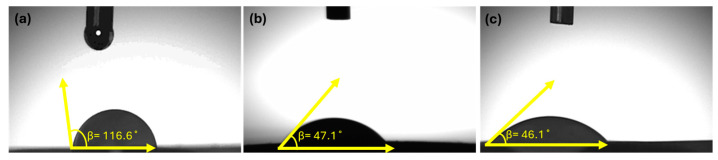
Average water contact angles of (**a**) NF-1/LL37, (**b**) NF-2/LL37, and (**c**) NF-3/LL37 samples were measured as 116.6, 47.1, and 46.1, respectively.

In this study, nanofibers were produced by blending PHB extracted from *Cereibacter sphaeroides*, commercial PHB, and marine-derived collagen, and loaded with the antimicrobial peptide LL37. Figure 6 presents the water contact angle (WCA) measurements used to assess the wettability of the developed nanofibrous scaffolds. The water contact angle (WCA) of the nanofiber with 0% bacterial PHB was 116.6°, indicating a hydrophobic surface. Increasing the bacterial PHB content to 25% and 50% reduced the WCA to 47.1° and 46.1°, respectively. These results demonstrate that higher bacterial PHB content significantly enhances hydrophilicity and improves the wettability of the nanofiber surface.

In the study of Sarıipek F.B., the water contact angle (WCA) of PHB nanofibrous scaffolds, which have a hydrophobic structure due to their crystalline regions, was measured as 91.49°. The water contact angle of PHB/CTS biocomposite nanofibrous scaffolds, fabricated by blending PHB with CTS, was measured as 44.39°, which is significantly lower than that of PHB. The hydrophilicity of CTS is primarily attributed to its hydrophilic amino and hydroxyl groups. As a result, when blended with PHB, CTS was observed to enhance the wettability of the fibrous scaffold [21]. The surface wettability of collagen nanofibers was investigated by Naderi Gharahgheshlagh et al. This test was performed on electrospun collagen nanofibers; however, due to the high hydrophilicity of this biopolymer, water droplets were rapidly absorbed, making it impossible to measure the water contact angle for the electrospun Kol-cotton, Kol/EPS1%-cotton, and Kol/EPS2%-cotton samples [22]. LL37 antimicrobial peptides, which possess an α-helical structure, exhibit amphiphilic properties; one end of such peptides is hydrophilic, while the other end has a hydrophobic region. This hydrophilic and lipophilic structure forms the basis of the antimicrobial function of this peptide [23].

### 3.2. Degradation of Nanofibers

Weight loss results exhibited that the cumulative degradation percentage of all samples ranged between 30% and 50%. The weight losses for NF-1, NF-2, and NF-3 samples at the end of 14 days were approximately 30%, 15%, and 50%, respectively. When the weight loss results are compared, the highest weight loss was observed in the 50% PHB_b_/Collagen sample, while the lowest was observed in the 25% PHB_b_/Collagen sample. In a study conducted by Mohammadalipour et al., the biodegradability of PHB and PCL polymers was investigated, and it was reported that a reduction in fiber diameter and crystallinity in scaffolds resulted in enhanced surface contact and easier PBS penetration, leading to higher biodegradability [24]. Similarly, in this study, a progressive decrease in nanofiber diameter was observed in the 0%, 25%, and 50% bacterial PHB/Collagen nanofibers. Consequently, the bacterial PHB/Collagen nanofiber containing 50% PHB_b_, which exhibited the smallest fiber diameter, showed the highest biodegradability. This observation is consistent with the thermal and gravimetric analyses, which demonstrated reduced stability of the nanofiber mats with increasing PHB_b_ content. It can therefore be inferred that a higher proportion of PHB_b_ may be advantageous for biomaterials requiring an accelerated biodegradation rate.

### 3.3. Biocompatibility and Cell Attachment on Nanofibers

**Figure 7:**
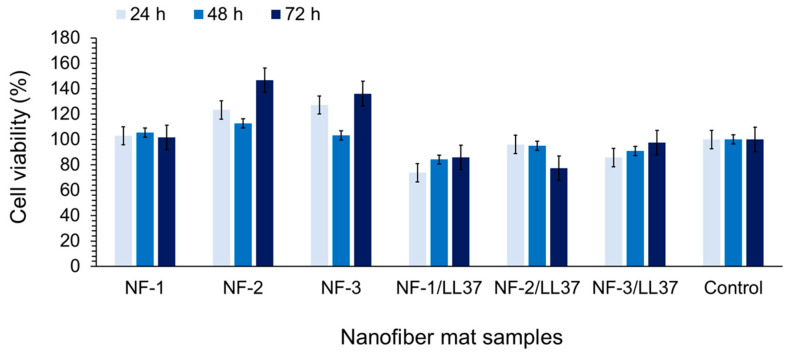
Effects of NF samples on cell proliferations after 24 h, 48 h, and 72 h treatment with NF sample extracts.

Fiber mats were incubated in cell culture medium for 24, 48, and 72 h. These extracts were then incubated with L929 cells previously seeded on culture plates for 24 and 48 h, and the cells were observed under an inverted microscope at 10× magnification. The effects of the extracts on cell proliferation were also evaluated after 48 h of incubation using an XTT cytotoxicity assay kit (Sartorius, Göttingen, Germany). Considering the proliferation of the control cell group as 100%, the proliferation rates of the cells incubated with the test groups were determined. As shown in Figure 7, cell proliferation values were found to be above 70%. The cell morphology after 24 h and 48h incubation with the blank NF sample extracts were presented in Figure S2a,b. Cell morphology after 24 h and 48 h incubation with the NF/LL37 samples extracts were presented in Figure S3a,b. Based on Figures S2 and S3, and taking cell morphology into account, it was concluded that the fiber mat samples were biocompatible. Cell images are presented as Supplementary Materials.

**Figure 8:**
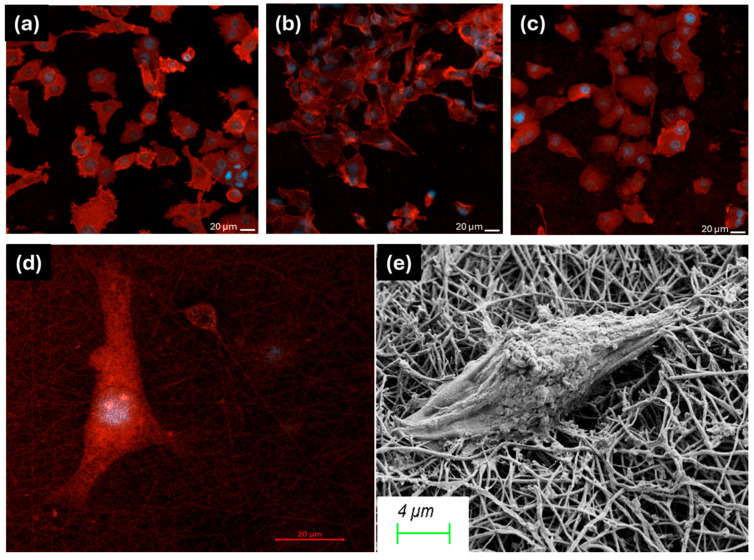
The CLSM images of keratinocyte cells attached to (**a**) NF-1/LL37, (**b**) NF-2/LL37, and (**c**,**d**) NF-3/LL37 samples. Nuclei were stained with DAPI (blue), and F-actin was stained using rhodamine–phalloidin. The cell attachment to the fiber matrix was also demonstrated by SEM (**e**).

SEM and confocal microscopy images of the PHB/Collagen nanofibers revealed the cell adhesion profiles on the nanofiber surfaces and demonstrated that cell attachment occurred on the non-toxic PHB/Collagen/LL37 nanofibers. As shown in Figure 8, keratinocyte adhesion was observed on the nanofiber mats containing 0%, 25%, and 50% bacterial PHB/Collagen/LL37, which are NF-1/LL37, NF-2/LL37, and NF-3/LL37, respectively.

### 3.4. Two-Dimensional Wound Healing Activity of Nanofiber Mats

**Figure 9:**
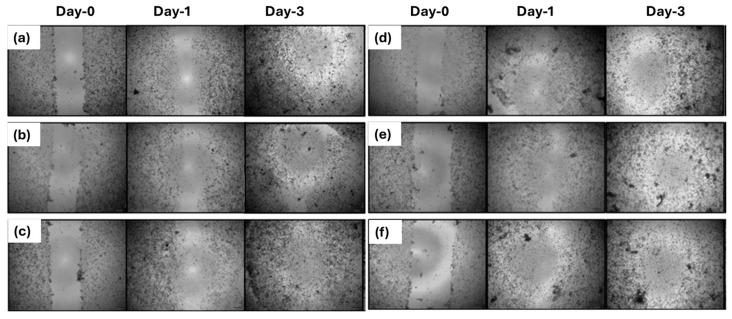
The microscopy views of the scratch assay. After wounding, the NF samples were put on the 2D wounds. After treatment for 1–7 days, the cells were monitored by an inverted microscope and the wound closure was measured; (**a**–**c**) are NF-1, NF-2, and NF-3, and (**d**–**f**) are NF-1/LL37, NF-2/LL37, and NF-3/LL37, respectively. On the 7th day, all the scratches were closed.

**Figure 10:**
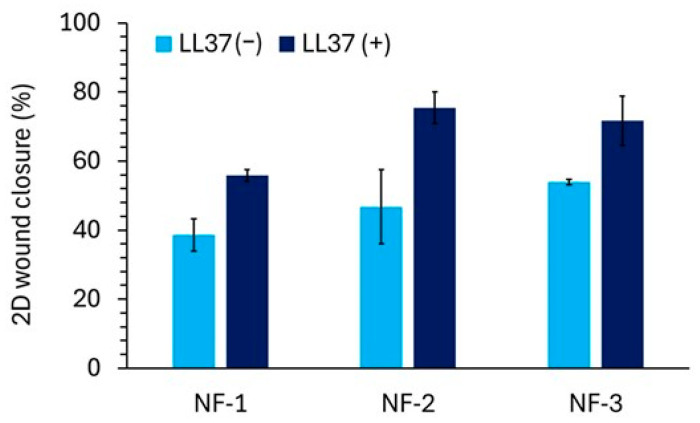
The wound closure percentage of the NF mat samples, measured by the distance of the closed scratch.

Wound closure percentages on days 1, 3, and 7 were calculated based on measurements from the microscopic images. Figure 9 shows the microscopic images of wound healing for nanofiber groups, including 0% bacterial PHB/Collagen, 25% bacterial PHB/Collagen, and 50% bacterial PHB/Collagen and those loaded with LL37 at the same ratios. When the values were compared, it was observed that wound closure occurred 15–30% faster in the LL37-loaded PHB/Collagen nanofibers (Figure 10).

Parallel to our results, a study by Fahimirad et al. demonstrated that PCL/PVA/CsLL37 and PCL/PVA/CsVEGF scaffolds showed superior wound healing performances compared to the PCL/PVA groups, highlighting the wound healing potential of the LL37 AMP [25].

### 3.5. Antibacterial Activity of Nanofiber Mats

**Figure 11:**
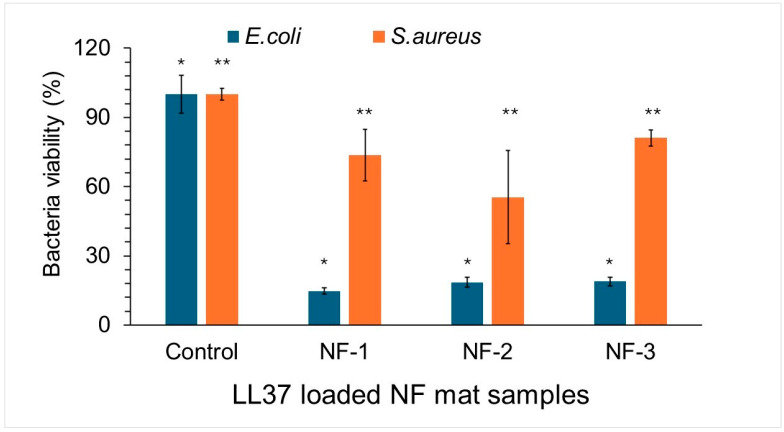
Viability of *E. coli* and *S. aureus* following treatment with NF/LL37 mats. The viability of both *E. coli* and *S. aureus* was significantly reduced compared to their respective untreated control groups (SPSS, Student’s *t*-test, *p* < 0.05). The symbol * indicates NF groups that exerted a significantly different percentage of *E. coli* viability compared to the untreated control, while ** indicates NF groups that exerted a significantly different percentage of *S. aureus* viability compared to the untreated control.

Antibacterial susceptibility tests are performed to determine the in vitro activity of an antimicrobial agent against a specific bacterial strain. *Staphylococcus aureus* and *Escherichia coli* were used as representative strains of common wound-associated bacterial infections [26]. Bacterial infections can lead to inflammation, increase exudate production, and negatively impact the wound healing process. Therefore, it is crucial for an ideal wound dressing to possess antimicrobial properties [27]. After treatment with NF/LL37 mats, the viability of *S. aureus* and *E. coli* was calculated as a percentage. Following exposure to fiber discs containing 55.6 µM LL37, the viability of *E. coli* decreased by 80–85%, whereas *S. aureus* viability was reduced by only 20–25% compared with the respective untreated controls (Figure 11). These results indicate that LL37-loaded fibers are more effective against *E. coli* than *S. aureus* under the tested conditions. According to Tavares et al. [28], LL37 demonstrates stronger antibacterial activity against Gram-negative bacteria such as *E. coli* and *P. aeruginosa*. This enhanced activity is explained by the peptide’s ability to electrostatically interact with lipid A and the phosphate groups of LPS residues, followed by its transition into an α-helical conformation that promotes a membranolytic mechanism. The stabilized helix lowers the energetic barrier for insertion and facilitates deeper penetration into the Gram-negative lipid bilayer. Based on the presented data, LL37 shows considerably greater activity against *E. coli* than *S. aureus*. The MIC for *E. coli* is 125 µg/mL, whereas a substantially higher concentration of 500 µg/mL is required to inhibit *S. aureus*. It is also shown that LL37 acts not only with greater potency but also with a faster antimicrobial response against *E. coli* compared to *S. aureus*. Consistent with these findings, our results also revealed a pronounced reduction in *E. coli* viability upon LL37 treatment, further supporting the notion that Gram-negative bacteria are more susceptible to LL37-mediated membrane disruption. Interestingly, Wang et al. [29] reported the opposite trend, showing that LL37 exerted stronger inhibitory effects on *S. aureus* than on *E. coli*, with a minimum bactericidal concentration (MBC) of 4 µM for *S. aureus* and a higher MBC of 32 µM for *E. coli*. Overall, these findings underscore that the carrier matrix influences peptide stability and release, directly affecting antimicrobial efficacy, and demonstrate that PHB/Collagen nanofibers represent a promising platform for the development of LL37-based wound dressings.

While the 2D wound healing and antibacterial performance of the LL37-loaded PHB/Collagen nanofibers was clearly demonstrated under in vitro conditions, it should be noted that the present study did not evaluate long-term stability or temperature-dependent effects on the activity of the scaffolds. These parameters are critical for the translation of nanofiber-based wound dressings into clinical applications, as storage conditions and physiological temperature fluctuations may affect both peptide stability and scaffold integrity. Therefore, future studies will focus on systematically investigating long-term stability, storage conditions, and temperature-dependent effects to better define the applicability of the developed materials.

## 4. Conclusions

In this study, biocompatible PHB/Collagen nanofiber wound dressing mats incorporating the antimicrobial peptide LL37 were successfully developed via electrospinning. The use of bacterial PHB derived from *Cereibacter sphaeroides*, in combination with commercial PHB and marine collagen, enabled the fabrication of stable nanofiber structures across varying PHB ratios. In vitro evaluations demonstrated that the nanofibers were non-cytotoxic to fibroblasts and keratinocytes, supporting their suitability as scaffolds for cell adhesion and growth. Furthermore, the nanofibers exhibited intrinsic antibacterial activity against *Staphylococcus aureus* and *Escherichia coli*, which was further enhanced by the incorporation of LL37. The LL37 loading applied in the fiber discs (5.56 nmol) was shown to be well-tolerated by dermal cells while promoting scratch closure and antibacterial effects.

Overall, these findings highlight the potential of PHB/Collagen nanofibers functionalized with LL-37 as advanced wound dressings that combine biocompatibility with antimicrobial efficacy. Future work should extend these in vitro observations to 3D tissue models and in vivo studies to validate their therapeutic performance in clinically relevant wound environments.

## Figures and Tables

**Table 1:** The parameters for the nanofiber manufacture process.

Nanofiber Types	PHB_(b)_ */PHB_(c)_ ** (*w*/*w*)	Collagen (w)	Solvent
NF-1: %0 bacterial PHB/Collagen	0 g PHB_(b)_/0.2 g PHB_(c)_	0.2 g	HFIP
NF-2: %25 bacterial PHB/Collagen	0.05 g PHB_(b)_/0.15 g PHB_(c)_	0.2 g	HFIP
NF-3: %50 bacterial PHB/Collagen	0.1 g PHB_(b)_/0.1 g PHB_(c)_	0.2 g	HFIP
NF-4: %75 bacterial PHB/Collagen	0.15 g PHB_(b)_/0.05 g PHB_(c)_	0.2 g	HFIP
NF-5: %100 bacterial PHB/Collagen	0.2 g PHB_(b)_/0 g PHB_(c)_	0.2 g	HFIP

* PHB_(bacterial)_, ** PHB_(commercial)._

**Table 2:** Functional groups of FT-IR spectra.

Functional Group	Wavenumbers (cm^−1^)
CH_3_	1380
CH_2_	1450
C-H	2976–2932
C=O	1724–1719
C-O	1280–1275
O-H	3436

## Data Availability

The original contributions presented in this study are included in the article/Supplementary Materials. Further inquiries can be directed to the corresponding authors.

## Supplementary Materials

The following supporting information can be downloaded at: https://www.mdpi.com/article/10.3390/polym17182486/s1, Figure S1: FE-SEM images of LL37 loaded NF samples; Figure S2a: Cell images after 24 h incubation with the blank NF sample extracts; Figure S2b: Cell images after 48 h incubation with the blank NF sample extracts; Figure S3a: Cell images after 24 h incubation with the NF/LL37 sample extracts; Figure S3b: Cell images after 48 h incubation with the NF/LL37 sample extracts.
