# Supplementary material for: Fabrication and In Vitro Evaluation of LL37-Loaded Electrospun PHB/Collagen Nanofibers for Wound Healing
Source: Polymers (Basel). 2025 Sep 15;17(18):2486. doi: 10.3390/polym17182486 (PMC12473663; doi:10.3390/polym17182486)
Supplement: Supplementary file 1 [file polymers-17-02486-s001.zip › polymers-3820561-supplementary.pdf]

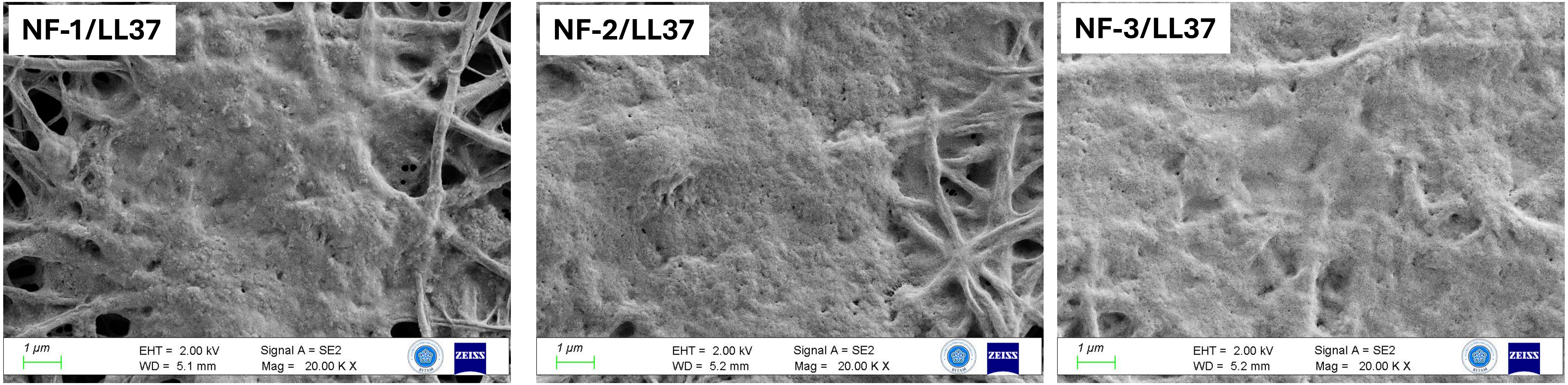

Figure S1. FE-SEM images of LL37 loaded NF samples

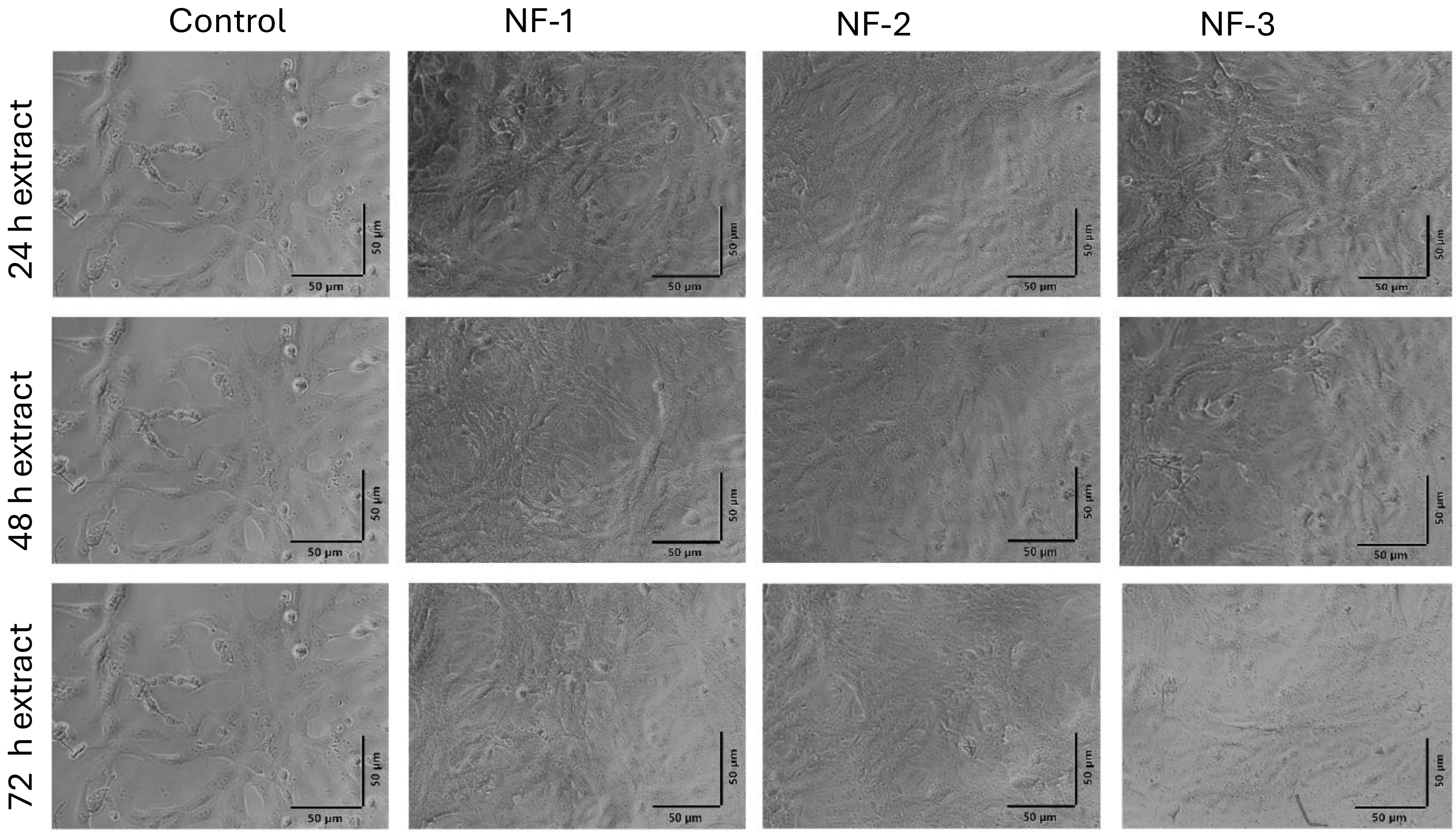

Figure S2a. Cell images after 24 h incubation with the blank NF sample extracts.

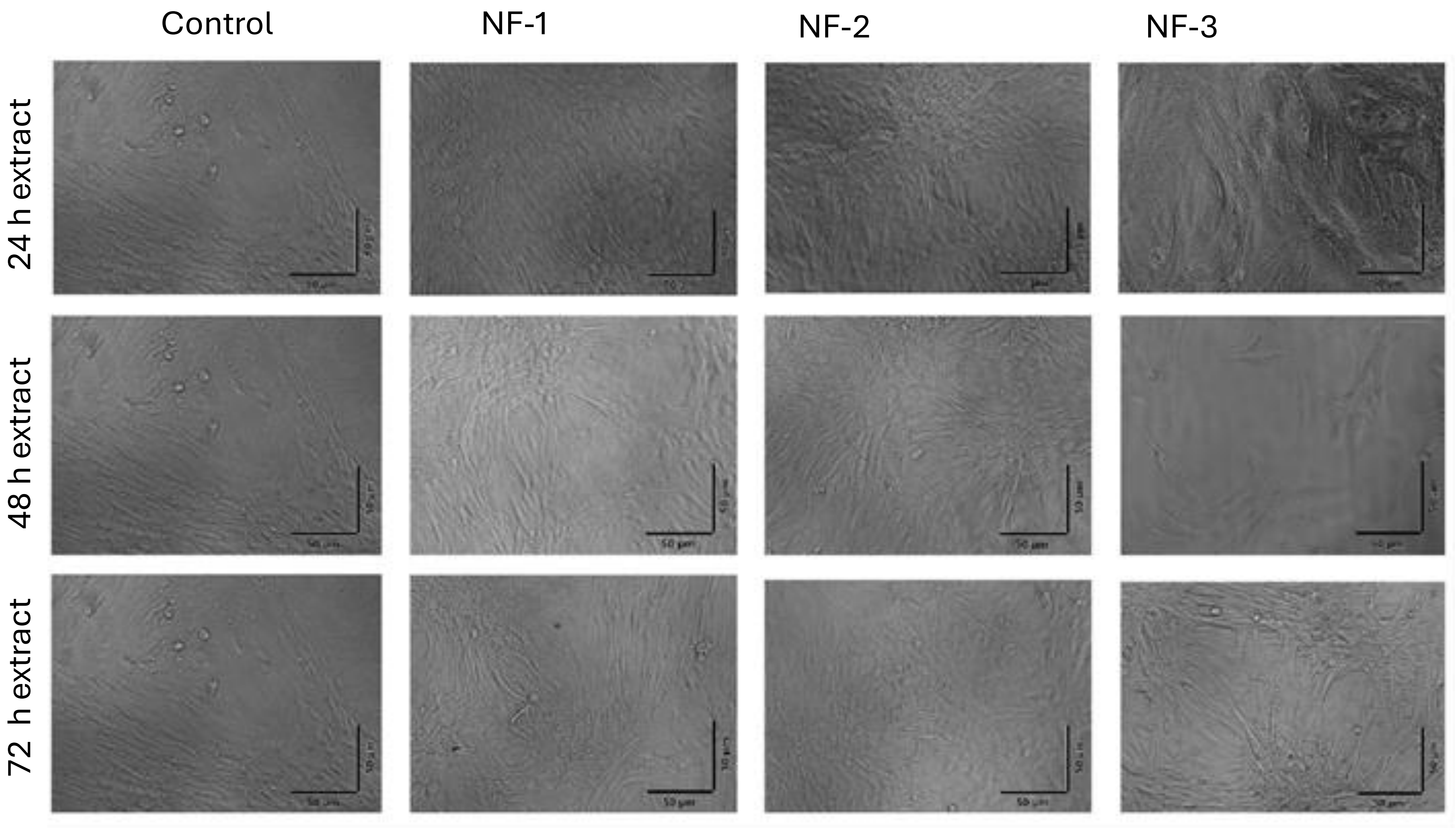

Figure S2b. Cell images after 48 h incubation with the blank NF sample extracts.

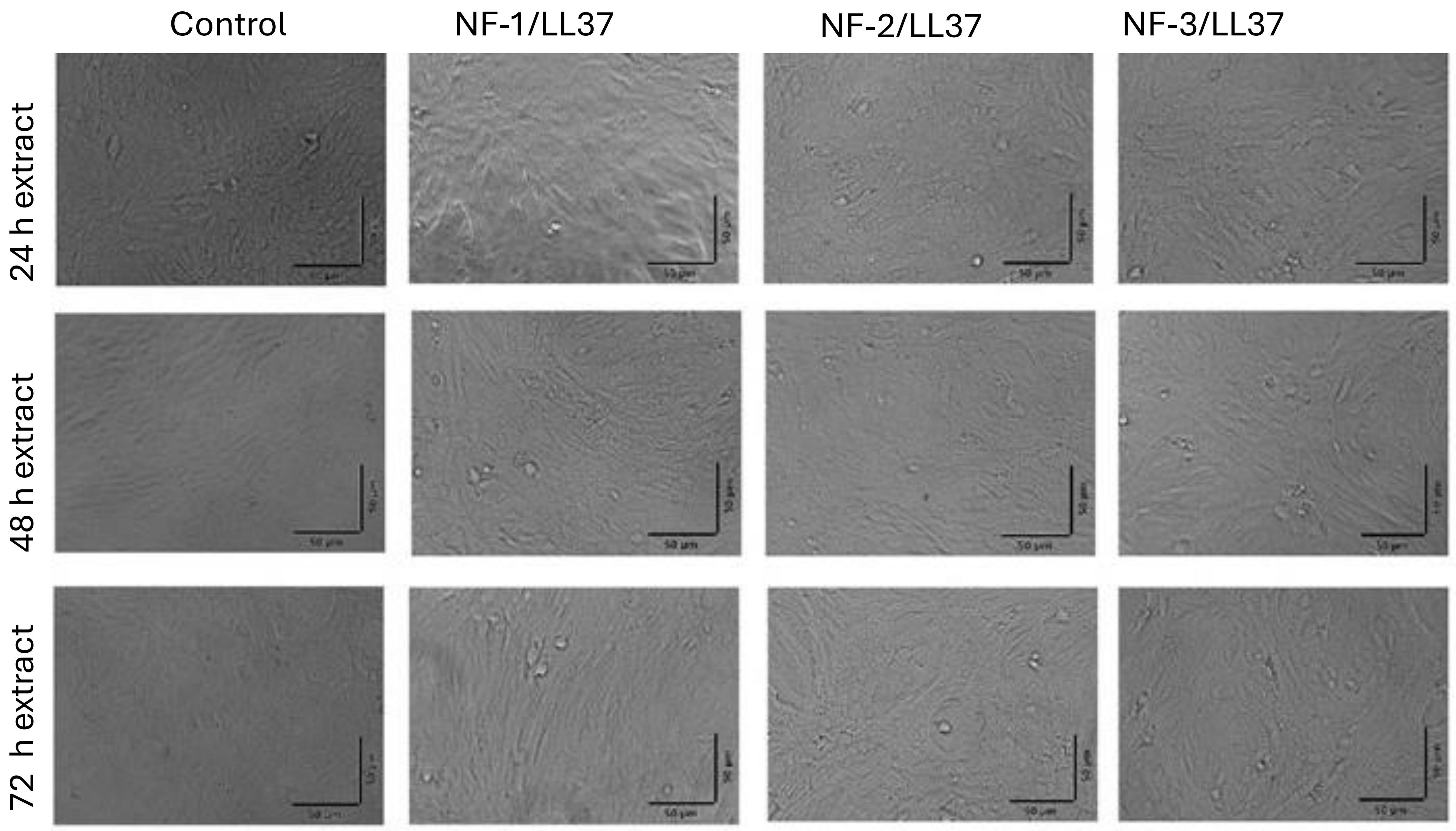

Figure S3a. Cell images after 24 h with the NF/LL37 sample extracts.

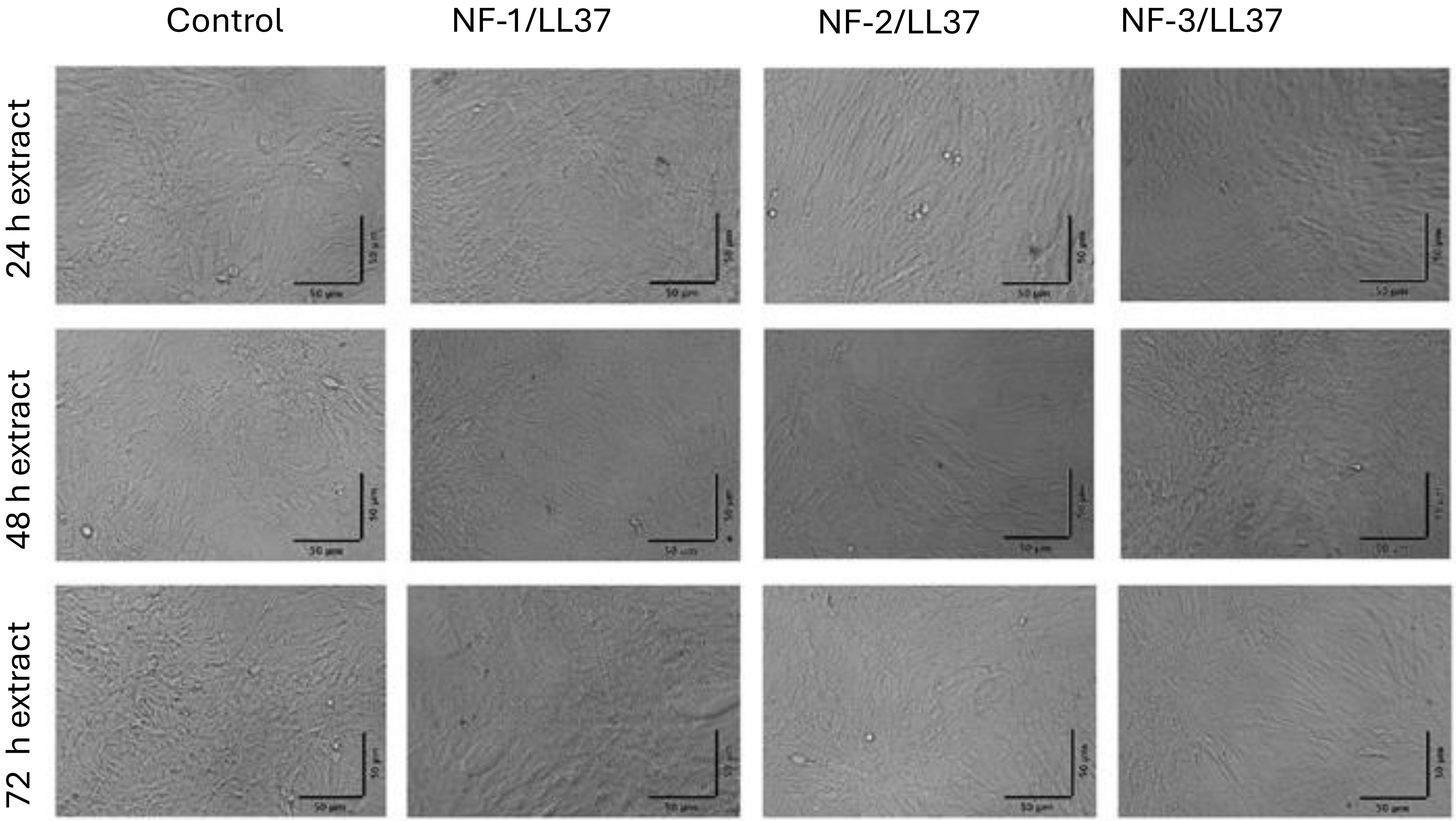

Figure S3b. Cell images after 48 h incubation with the NF/LL37 extracts.
